# HIV Prevention in Adolescents and Young People in the Eastern and Southern African Region: A Review of Key Challenges Impeding Actions for an Effective Response

**DOI:** 10.2174/1874613601812010053

**Published:** 2018-07-19

**Authors:** Kaymarlin Govender, Wilfred G.B. Masebo, Patrick Nyamaruze, Richard G. Cowden, Bettina T. Schunter, Anurita Bains

**Affiliations:** 1 Health Economics and HIV and AIDS Research Division, University of KwaZulu-Natal, Durban, South Africa; 2 School of Applied Human Sciences, University of KwaZulu-Natal, Durban, South Africa; 3 Department of Psychology, Middle Tennessee State University, Murfreesboro, United States of America; 4 *UNICEF, Eastern and Southern Africa Regional Office, Nairobi, Kenya*

**Keywords:** HIV, Prevention, Adolescents, Young people, Africa, SRH services

## Abstract

The global commitment to ending the AIDS epidemic by 2030 places HIV prevention at the centre of the response. With the disease continuing to disproportionately affect young populations in the Eastern and Southern African Region (ESAR), particularly adolescent girls and young women, reducing HIV infections in this group is integral to achieving this ambitious target. This paper examines epidemiological patterns of the HIV epidemic among adolescents and young people, indicating where HIV prevention efforts need to be focused (*i.e.*, adolescent girls and young women, adolescent boys and young men and young key populations).

Key innovations in the science of HIV prevention and strategies for dealing with programme implementation are reviewed. The paper also discusses the value of processes to mitigate HIV vulnerability and recommends actions needed to sustain the HIV prevention response. Stemming the tide of new HIV infections among young people in the ESAR requires an amplification of efforts across all sectors, which will safeguard past achievements and advance actions towards eliminating AIDS as a public health threat.

## 1 INTRODUCTION

**Fig. (1):**
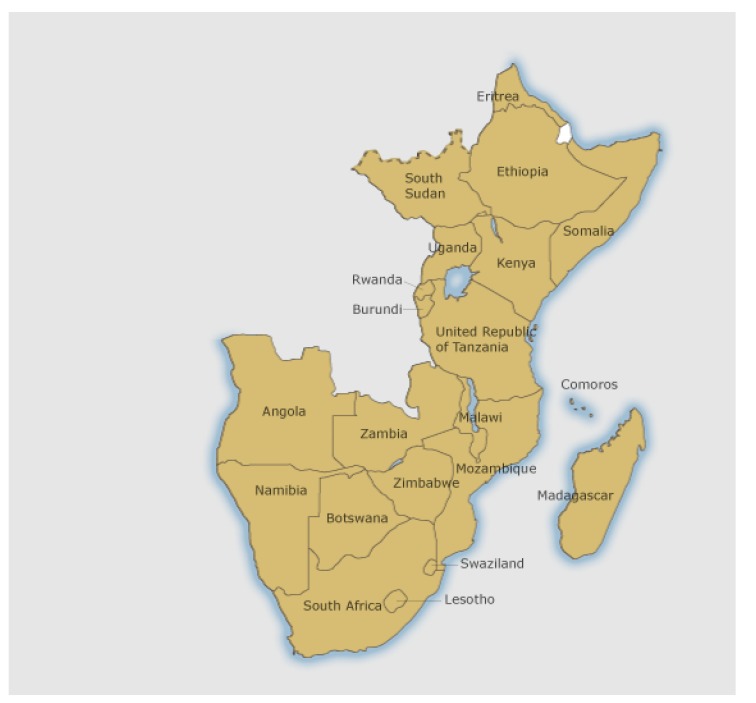
Map showing countries in Eastern and Southern Africa Region [1].

Substantial progress has been made in the AIDS response under the Millennium Development Goals framework. Yet, adolescents and young people^1^ are still heavily affected by the disease, accounting for 37% of all new HIV infections in 2017 and 15% of all people living with HIV [1, 2]. While the overall number of AIDS-related deaths declined by 48% between 2005 and 2017, AIDS-related deaths among all adolescents and young people increased by 50% to approximately 55,000 deaths [2]. The greatest number of new infections in the Eastern and Southern African Region (ESAR) (Fig. **1**) occur among adolescents and young people [3]. In this region, access to HIV and sexual and reproductive health (SRH) services is limited and social stigma and human rights violations are widespread [4, 5].

If the target of reducing the number of new HIV infections by 75% by 2030 is to be achieved [6], amplified HIV prevention efforts are necessary. Accordingly, this paper outlines some of the key challenges impeding efforts to reduce the number of new HIV infections among adolescents and young people in the ESAR. The paper begins by describing epidemiological patterns in the HIV epidemic among adolescents and young people, populations where HIV prevention efforts are necessary, current approaches to HIV prevention programming and their limitations. Further, consideration is given on the broader developmental priorities for sustaining the HIV prevention response, including social protection programming to mitigate HIV vulnerability, innovative ways in which to finance HIV prevention activities, and areas where civil society involvement can be strengthened.

## 2 EPIDEMIOLOGICAL PATTERNS OF HIV IN THE ESAR

Recent evidence suggests that the global burden of HIV in young populations is predominant in the ESAR. In 2017, the estimated number of new infections among young people in the ESAR aged between 15 and 24 years was 300,000, with the number of AIDS-related deaths in this age group approximately 36,000. [2].

**Fig. (2):**
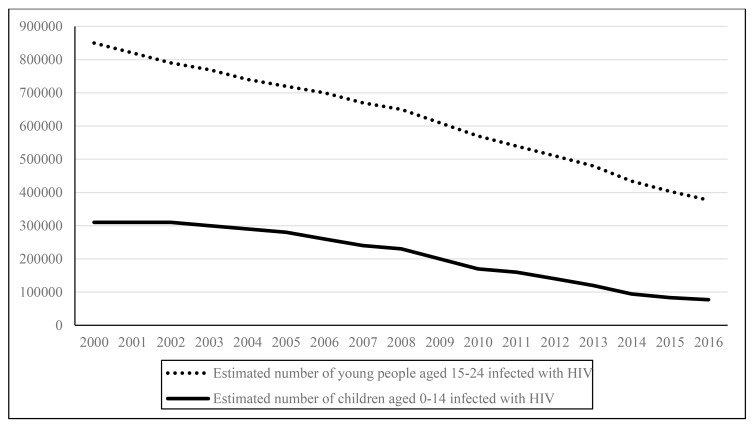
Estimated number of new HIV infections among children and young people in the ESAR, 2000-2016 [2].

Examining the HIV infection rates among children and young people in the ESAR over a period of 16 years (Fig. **2**), sub-epidemic age-related patterns indicate that while there are declines in new infections in both cohorts, new infections among young people are comparatively higher. Almost two in five (300,000 out of 790,000) new infections in the ESAR in 2015 were among young people between 15 and 24 years old, and one in five (130,000 out of 790,000) among adolescents between 15 and 19 years of age [2].

WHO defines people between 10 and 19 years of age as adolescents, and those between 10 and 24 years of age as young people.

**Fig. (3):**
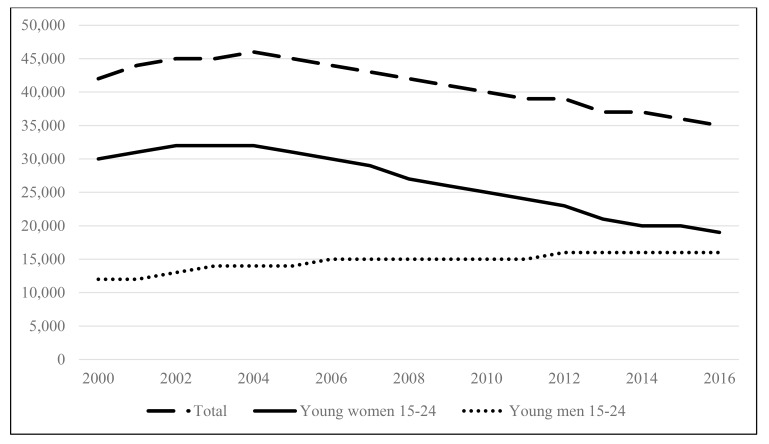
HIV-related deaths among young women and young men in the ESAR, 2000-2016 [2].

**Fig. (4):**
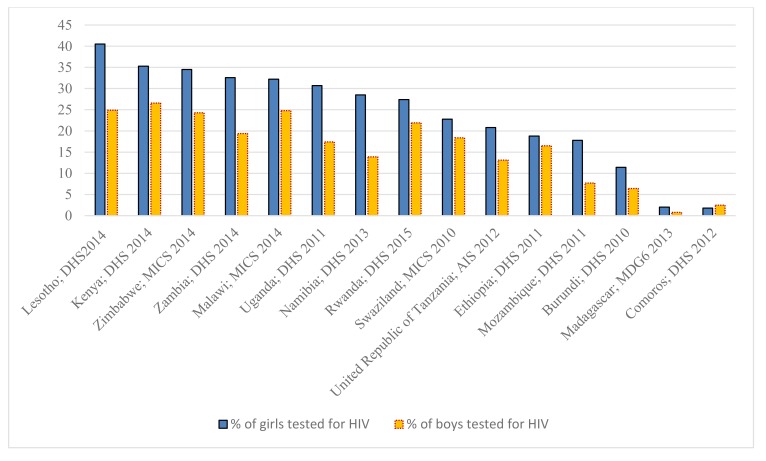
Percentage of adolescent girls and adolescent boys (15 to 19) tested for HIV and received results in the last 12 months, in the ESAR, 2010-2015 [2, 7].

Although AIDS-related deaths in the ESAR among adolescents and young people have generally been decreasing, the mortality rate remains high among adolescent girls and young women and is increasing among adolescent boys and young men (Fig. **3**). A closer look at the data on AIDS-related deaths points to age and gender patterns. The 2017 data indicated that mortality levels were higher among young women 20 to 24 years old (21,000) as compared to their male counterparts (7,300), yet were lower for adolescent girls (7,900) than for adolescent boys (9,000) [2]. Higher mortality rates for adolescent boys (as compared to adolescent girls) may be due to undiagnosed cases of HIV in this vertically infected population. This means that although more women acquire HIV, more men die of AIDS. Part of the explanation could be that women have more interactions with health facilities (pregnancy, taking children for immunisation) so have opportunities to be tested and treated. Also, it could be that older adolescent boys and young men are just not being captured in the HIV response. For example, adolescent boys and adolescent girls differ in their HIV status knowledge. Demographic Health Surveys (DHS) data suggest that more adolescent girls have had an HIV test and are aware of their HIV status as compared to adolescent boys (Fig. **4**).

Given the complex factors that drive HIV risk among young people in the ESAR, current epidemiological evidence is limited in several ways. First, there are a number of methods to measuring HIV incidence (*e.g.*, cohort estimation, mathematical modelling, inference incidence from antenatal clinic data, laboratory tests, a combination of HIV testing algorithms), each with benefits and limitations [8]. More recently, these methods have been complemented by phylogenetic and geospatial epidemiology [8, 9]. Given that some of these methods employ newer technologies, more expertise and resources are required for implementation especially in low resourced settings. In addition, most national surveillance systems are not fully equipped to implement location-based approaches at sub-national or at health facility levels [8], which results in a lack of context-specific data (by age and gender) to inform locally-based programming. The challenges in tracking the epidemic at local levels is further compounded by low rates of HIV testing [5]. Second, there is a lack of data on younger adolescents (those below 15 years) because of challenges in getting valid (and ethically sanctioned) information. Understanding issues of sexuality and sexual risk in young children is important for informing HIV and sexual and reproductive health (SRH) risk mitigation strategies (*e.g.*, delaying sex, using condoms, regular health screening) before they get older and more exposed to the risk of HIV infection. In addition, when one takes into consideration adolescent key populations (*e.g.*, adolescent girls engaged in sex work, men who have sex with men, people who inject drugs and the lesbian, gay, bisexual, transgender and intersex [LGBTI] community), there is much debate on accuracy of population size estimates at country level, which ultimately has a bearing on resource allocations for HIV programming [5]. Third, self-reported behavioural data (when it is available) is plagued by measurement issues. Coupled with small sample sizes, especially among younger cohorts, the generalisability of such findings is questionable. Scant data also exists on the broader health and social issues of young populations (*e.g.*, mental health, developmental adjustments to social environments and decision-making skills). Fourth, there are few efforts devoted to interrogating conflicting data reported on adolescents and young people. For example, in Botswana, condom use is reportedly high and exceeds national targets for both sexes, yet adolescent HIV incidence rates and early pregnancy rates are increasing [10].

There is a clear need for triangulation of different data sources to obtain a well-defined picture of the epidemic and trends over time at country and regional levels. HIV data that is collected should be integrated with data on the prevalence of pregnancies and access (SRH) facilities. Data shows that antenatal attendance rates in the ESAR are relatively high at 77% [11-14], with some clients being older adolescent girls and young women. This finding is not surprising given that adolescent pregnancy rates in the ESAR are among the highest in the world, ranging from 19% to 29% in Lesotho, Kenya, Malawi, Mozambique, South Africa and Zambia [11-14]. Finally, data gathered in different settings need to be extended to planning interventions, including target setting, monitoring and advocacy.

## 3 YOUNG POPULATIONS THAT REQUIRE TARGETED HIV PREVENTION INTERVENTIONS

### 3.1 Adolescent Girls and Young Women

In 2017, HIV prevalence among young women (15 to 24 years) was double that of young men in the ESAR (3.4% compared to 1.6%), although in some countries the disparity between women and men was even greater [2]. In the ESAR, the gender differentiation of the HIV epidemic appears to start at an early age. For example, in Swaziland, among 15- to 19-year old adolescent girls and adolescent boys, 6,900 and 2,500 were infected, respectively [2]. This discrepancy was higher in Lesotho, with 8,400 adolescent girls between 15 and 19 years of age infected compared to boys of the same age cohort (3,700) [2]. The 2017 data for the ESAR indicates that 98,000 of new HIV infections occurred among adolescent girls aged 15 to 19 years, and 110,000 new HIV infections occurred among young women aged 20 to 24 years [2]. The number of 15- to 19-year-olds living with HIV in the ESAR was as high as 490,000 among adolescent girls and 270,000 among adolescent boys [2]. Despite comprising 10% of the population, young women aged 15 to 24 years accounted for 26% of new HIV infections in the ESAR in 2017 [2]. These data point to the high-level vulnerability of adolescent girls and young women.

The inability to negotiate for condom use and vulnerability to multiple and concurrent sexual partnerships, age disparity in sexual relationships, transactional sex, as well as gender-based violence (GBV) are some of the drivers of these trends in the region [15]. Country-level data reveals that age-disparate sexual relationships are common, with many adolescent girls being infected by young men who are at least five years older than they are. Data from Swaziland, Lesotho and Zambia point to a significant association between a young woman’s HIV status and the number and age of her partners [11, 13, 16]. In Swaziland, for instance, a young woman with more than one older partner is three times more likely to be HIV positive then those not having older partner then those not having older partner [16]. Adolescent girls who often engage in such a relationship for economic and other material reasons usually have very limited possibilities to negotiate for safe sex [17]. A recent phylogenetic analysis of the HIV virus in KwaZulu-Natal, South Africa [18] found evidence of the high-risk nature of age-disparate sexual relationships, while another study in the same region did not support the relationship between age-disparate sex and HIV acquisition in adolescent girls and young women [19]. Clearly, more research is needed to understand contextual factors that influence the choice of sexual partners and how these choices are associated with HIV risk.

### 3.2 Young Key Populations

Young Key Populations (YKP) in the ESAR face significant barriers to accessing HIV and SRH services. Key populations, which refer to sex-workers, people who inject drugs, the imprisoned population, and the LGBTI community, are extremely diverse groups. Young people, who are also members of key populations, are a frequently neglected subset of this group. YKP, which often include adolescents, face additional legal, policy and social barriers to accessing HIV and SRH services [5].

While regional and international treaties guarantee a number of rights to SRH services for young people, including those who identify as members of key populations, there are weaknesses in the legal environments of many countries in the ESAR. For young people who belong to one or more YKP, age-related barriers to accessing HIV and SRH services are further compounded by in-country laws and policies that criminalise their sexual activities, identities and behaviours [20, 21]. Designation of sexual activities and identities as illegal impacts access to healthcare, which can increase vulnerability to HIV.

YKP are especially vulnerable to risks associated with limited access to quality SRH services through widespread discrimination, stigma and violence, which are often compounded by vulnerabilities (*e.g.*, youth-power imbalances in relationships), and, sometimes, alienation from family and friends [22]. Young female sex workers, for example, are at risk of unwanted teenage pregnancies and sexually transmitted infections [23], while young transgender and lesbian women are sometimes the targets of so-called ‘corrective’ rape [24]. Young prisoners are often exposed to enduring sexual abuse, which goes largely unrecognised and unpunished [25]. Despite exposure and vulnerability to these circumstances, YKP tend to be reluctant to seek support from healthcare providers [20, 26, 27].

YKP are rarely involved in policy development or consulted during law reforms. As a result, inadequate government funding is directed towards research, prevention, treatment and care programmes that are responsive to their needs. Given the punitive legal and socio-cultural contexts in the region, which are intolerant to the diversity of identities and sexual practices [20, 26, 27], more work needs to be done to document experiences of such populations. This would strengthen legal and policy environments that help accelerate access to HIV and SRH services, as well as reduce human rights violations and health risks within these groups. Progressive steps have been made towards achieving this, with the Global Fund now requiring grant applicants consult KP when developing their applications for funding [24].

### 3.3 Boys and Young Men

HIV prevalence rates remain much lower among adolescent boys and young men compared to their female counterparts, particularly before the age of 20 years [2]. Owing to these low prevalence rates, inadequate attention has been directed towards HIV prevention and treatment programmes for this population [28, 29]. While adolescent boys might not be drivers of HIV infections among girls and young women, data shows that HIV incidence rates among young men steadily increase between 20 and 30 years of age [2, 3, 5]. This is attributed to the internalisation of traditional masculine values and misogynistic gender attitudes espoused in the region, which are closely linked to sexual practices that predisposes young men to the risk of HIV infection (*e.g.*, unprotected sex, multiple and concurrent sexual partnerships, sex while under the influence of alcohol, and gender-power relations linked to sexual violence) [30-33]. Previous research indicates that these normative behaviours are developed during identity formation stages in early adolescence [31, 34-37]. Therefore, understanding masculine identities associated with transitioning to adulthood that influence sexual risk behaviours need to be a central part of HIV prevention intervention.

In most African countries, SRH services are essentially considered women’s domain, thereby, neglecting men. Adolescent boys and young men are less likely to know their HIV status and utilise HIV services [2, 7, 29]. Therefore, engaging in approaches to promote the health of adolescent girls and young women requires efforts that cultivate a sense of gender equality among men at a young age [32, 33]. Engaging boys and young men in HIV prevention interventions requires a generalised understanding of the health-seeking behaviours of young men. Evidence suggests that well-implemented and developmentally appropriate behaviour change communication programmes, which promote gender equality, can potentially influence attitudes and behaviours of young men [34]. However, programming of this nature needs to consider age and cultural differences and similarities across sub-populations of young African men.

Accordingly, prevention programming should factor in processes to understand masculinities and HIV risk in different contexts to more clearly inform the development of locally-based gender-sensitive interventions [35]. Gender-sensitive interventions (*e.g.*, Stepping-Stones, livelihood strengthening, peer networks) [36, 37] require intensive and prolonged engagement with men. While there are no swift solutions to changing men’s behaviours, encouraging adolescent boys and young men to participate in HIV prevention activities at an early age is essential for not only promoting healthy masculinities, but also the health of adolescent girls and young women [28].

In addition, there is a need to increase the participation of young men and their uptake of HIV services, including Prevention of Mother to Child Transmission (PMTCT), family index testing, and pre-exposure prophylaxis (PrEP) for discordant couples [38]. Voluntary Medical Male Circumcision (VMMC) is seen as one key entry point for HIV prevention among adolescent boys and young men [39]. Although the number of circumcised males has tripled in the last two years, seven out of ten males have not yet had the chance to be circumcised in the 14 priority countries in the ESAR [33]. The demand for VMMC services is greater among adolescent boys under the age of 19 years in the ESAR [33, 40, 41]. VMMC performed before the age of sexual debut has maximum long-term impact on reducing individual HIV risk, and, consequently (if scaled up), reduces the risk of transmission in the population [42]. Offered as a comprehensive package, adolescent VMMC can potentially increase public health benefits and offers opportunities for addressing gender norms. However, additional research is needed to assess whether current VMMC services address the specific needs of adolescent clients, to test adapted tools, and to assess linkages between VMMC and other adolescent-focused HIV, health and social services.

## 4 CHALLENGES ASSOCIATED WITH HIV PREVENTION PROGRAMMING AND RESEARCH

### 4.1 Multifaceted and Cascaded HIV Programming

The science of combination approaches to HIV prevention, including VMMC, comprehensive sexuality education and access to SRH services, is considered as an optimal response and is meant to reflect a coordinated implementation of behavioural, structural and biomedical strategies targeting a particular population [43]. While this approach is conceptually sound, success of such strategies are limited by underlying gender-power dynamics, insufficient sexuality education and the absence of quality health services [44]. Even when interventions are introduced, they are often applied asynchronously or combined without synergy [27, 45].

The importance of recognising the heterogeneity of a single population and the need for differentiated HIV prevention approaches [46] has prompted some researchers to consider interventions from an HIV prevention cascade perspective. The prevention cascade guides the design and monitoring of HIV prevention programmes through a multi-pronged approach that includes demand-side interventions, supply-side interventions and adherence interventions [47, 48]. Demand-side interventions are aimed at improving risk perception and awareness and acceptability of prevention approaches [48]. Supply-side interventions are aimed at making prevention products and procedures more accessible and available [48]. Adherence interventions support ongoing adoption of prevention behaviours [48].

The advantage of the cascade approach is that it identifies population-level constraints to adopting proven biomedical, behavioural, and structural strategies for HIV prevention [49], while also serving as a useful tool for planning interventions and monitoring gaps in HIV prevention. So far, the cascade approach has been successfully used in PMTCT and in monitoring HIV treatment for viral load suppression [49]. The challenge for HIV prevention programming is addressing the behavioural and structural factors that influence the uptake of and adherence to biomedical regimens [49]. These include issues such as acceptability, demand, perceived efficacy for self-care, stigma and discrimination, and life challenges (*e.g.*, poverty, violence) that may impede an individual’s ability to adopt and adhere to prevention regimens [48,] (Table **1**).

An illustration of these challenges has been aptly demonstrated with the introduction of antiretroviral medicines (ARVs), both to prevent HIV acquisition (PrEP) and to minimise onward transmission. This new biomedical prevention modality has introduced renewed hope for eradicating HIV [50], with studies showing that daily oral PrEP is safe and efficacious among populations at substantial risk of becoming infected with HIV, including young men and women [51]. However, low adherence levels to PrEP among adolescents in pilot and experimental interventions raise concerns about its feasibility as a public health strategy [50, 51]. Issues associated with medication adherence include inadequate use, lack of instruction comprehension, influence of partner beliefs, unfavourable side-effects, low HIV risk perceptions and living in poor socioeconomic conditions [3, 52]. In addition, issues related to the need for criteria on who should be eligible, length of eligibility or treatment, and the cost effectiveness of scaling-up treatment are important considerations.

As indicated, biomedical HIV prevention offers much promise as a component of comprehensive prevention strategy packages. However, this method poses a number of challenges for programme implementers. How can combination prevention programmes be tailored for specific contexts? Young people have very different HIV risk profiles, HIV service access needs, and socio-developmental challenges relative to sexual decision-making compared to adults older than 24 years. Further, these factors vary widely among young people, especially based on age and gender. Therefore, there is no universal, ‘one-size-fits-all’ approach to delivering HIV services for young people.

Ongoing complex, multi-component intervention trials and programmes that are being implemented in different settings can provide the necessary guidance on the type and degree of combinations appropriate for different populations in their specific context [53]. An example is the Determined, Resilient, Empowered, AIDS-free, Mentored, and Safe women (DREAMS) programme, which aims to reduce HIV infections among adolescent girls and young women (aged 15 to 24 years) by 40% in ten ESAR countries [54]. Although this modality of prevention programming is promising, implementation often varies conditionally based on location (*e.g.*, high HIV prevalent zones) and strength of community systems. Therefore, interventions need to be coupled with ongoing situational analyses to improve HIV delivery platforms that ensure these interventions reach the intended populations. Evidence from both scientific and operational research needs to be collated in order to shape the policy environment, which will facilitate large-scale implementation with high quality and intensity [48].

### 4.2 Issues of Consent Related to Research and Access to HIV and SRH Services

Research on HIV prevention among adolescents and young populations is needed to understand the determinants of specific patterns of sexual behaviour, predictors of sexual activity debut, and the health and psychosocial needs that result from these issues [55]. Undertaking such research, however, requires appropriate ethical guidelines to ensure these vulnerable groups are adequately protected while undertaking research that is relevant and appropriate to their needs [27]. Current ethical and legal standards in most countries in the ESAR make it difficult to conduct health research with people under the age of 18 years [56]. People younger than 18 years of age are often legally designated as incapable of consenting to research participation [55], with consent only possible through parents or legal guardians [57]. The current approach results in a lack of adequate data, and, when data becomes available, the indicators of HIV risk and sexual behaviours are likely biased.

Recently, some progress on biomedical HIV research with children and adolescents has been made, but there still exists a reluctance to involve adolescent participants in clinical research in many countries because of the complex legal and ethical requirements [57, 58]. These complexities include researchers studying illegal behaviours (*e.g.*, underage or other forms of criminalised sex, intravenous drug use, or sex work), researchers needing to provide access to HIV and SRH health services, which may be illegal in some countries, and minors having privacy expectations regarding their health status as part of ongoing studies. Researchers also need to ensure field staff are adequately trained to conduct research with minors and have the necessary medical and psycho-social mechanisms in place to manage adverse events that may be encountered during research [58]. Given these complexities, there is a need to simultaneously ensure protection of minors from any form of harm when undertaking research that is highly beneficial to them.

A related concern is the obstacles faced by adolescents in accessing HIV and SRH services and information [59]. In many ESAR countries, the legislature is often contradictory. Usually, there are minimum ages of consent for sex (typically 15 or 16 years of age), but also minimum age restrictions for accessing SRH services independent of parents (usually above 18 years when a potential client is recognised legally as an adult). In some countries, the minimum age for sex (in teens) is contradicted by statutory definitions of a ‘child’ and associated laws for child protection [60]. Although parents have the legal right to make health decisions on behalf of their children, this can hinder their child’s access to and threatens their confidentiality when seeking SRH services [3].

In Fig. (**4**), there are low rates of HIV testing among adolescents in some countries, with less than 5% reported in Madagascar and Comoros. One explanation for this is that the legal age of consenting to SRH services in some countries (*e.g.*, HIV testing and counselling and contraceptive access) is unclear or unspecified. Consequently, service providers may apply “age-appropriateness” of consent to HIV testing at their discretion, and there is likely to be confusion about the situations and treatment types for which minors do and do not require parental consent [60]. Therefore, it is imperative that countries clearly set out the minimum age of consent to sexual activity and ensure that this aligns to the age of consent to SRH services, including contraceptives. The criminalisation of consensual sexual activity among minors in some countries in the ESAR also hinders access to SRH services, such as preventative measures (*e.g.*, contraception) [60]. The low rates of HIV testing are also attributed to social stigma, concern over being HIV positive, risk-naivety and a lack of awareness about testing facilities [61]. Those who are unaware of their HIV positive status are unlikely to seek antiretroviral treatment, and, are predisposed to transmit HIV unintentionally [62]. In addition, inadequate management of learner pregnancies often restricts access to education and health [63]. Only half of the countries in the ESAR have legislation and policies on the management of learner pregnancy and re-entry to school [63]. Excluding learners because of their pregnancy limits their access to education and health, and perpetuates gender inequality [63].

While HIV and SRH information and services need to target those with the highest risk of infection, such interventions should be designed on the premises that young people are capable of understanding information, appreciating risks and making informed decisions about their SRH [59]. Further, age-based criteria for independent access to health services needs to be standardised across settings, with consent also taking into account developmental and contextual factors that relate to the vulnerability of young people, which are not necessarily chronological. Consequently, it is important to accommodate the evolving capacities of young people, especially minors, with procedures in place that allow service providers to assess consent capabilities of minors. This needs to be accompanied by supportive social change mechanisms and engagement of service providers who can offer friendly services.

## 5 MITIGATING VULNERABILITY AND SUSTAINING THE HIV PREVENTION RESPONSE

### 5.1 Keeping Social Protection on the Agenda

Social protection is a mechanism for addressing the structural barriers experienced by the poor and vulnerable. More specifically, HIV-sensitive social protection addresses the socio-economic determinants of vulnerability, where financial protection programmes support access to affordable quality services, and country policies and legislation to uphold the rights of the most vulnerable people in high HIV prevalence contexts [64].

Many countries in the ESAR provide financial protection (*e.g.*, cash transfers), which has been influential in reducing poverty [65, 66]. Cash transfers can increase household income and the affordability of healthcare and nutrition, which ultimately improve health outcomes [67]. Several studies have shown the benefits of cash transfers and other economic incentives for preventing HIV among adolescent girls and young women [68, 69], which have also been used successfully to encourage safer sexual practices in the ESAR [65, 67]. Cash transfers are more likely to have an effect on reducing HIV if they can increase school attendance or meet survival needs, thereby deterring adolescent girls from engaging in transactional and age-disparate relationships [66].

While cash transfers are proving to be a key component of interventions aimed at HIV prevention, available evidence supports augmenting financial support with social support from parents and teachers, free education and support services rather than cash alone [70]. In a South African-based study from 2009-2012, past-year incidence of sexually risky behaviour was higher among both adolescent girls and adolescent boys who received cash transfers alone, as compared to those that received a combination of cash, free education, and parental monitoring. The likelihood of engaging in unprotected sex was reduced by approximately 40% when adolescents between 12 and 18 years of age were provided a combination of social protection factors, including access to education, parental monitoring and sensitive clinical care. When cash, free education and parental monitoring were combined, condom use at last sex increased from 47.1% to 60.8% among young men, and from 50% to 64.3% among young women. Testing for HIV also increased from 46% to 56% among young males and from 63% to 73% among young females [70].

Although evidence points to the need for a combination approach to providing social protection [71], these approaches require more well-developed evidence that focuses on outcomes from diverse contexts. Social protection systems need to appreciate the unique and nationally specific needs of adolescents and young people in the ESAR [72]. However, funding for social protection remains a significant challenge for many countries in the ESAR, as resources for investing in social protection programmes are scant and depend on donor projects [73]. The challenge of ensuring adequate and sustainable financing for social protection is compounded by economic uncertainties and competing agendas in these countries, resulting in reluctance among donors to enter into long-term funding commitments [73]. Despite these issues, there is a need to move away from short-term project funding towards providing longer-term domestic resources for combined social protection programmes.

Such programmes should move beyond cash alone to in-kind components combined with care and building individual capabilities in young people. Social protection programmes for the poor should also do away with categorically targeting the most vulnerable groups and rather focus on the entire community [70, 74, 75]. Simply, social protection programmes have to be linked to basic social services of cash, care, health, nutrition, education and protection. Locally-based implementers should lean on the current evidence to design and implement intervention in their communities. These programmes also require coherence and integration into stronger social welfare policy that extend beyond safety nets [69] by drawing on the technical and financial capacities of governments. The Government of Zambia, which uses its national budget for its social protection programme, is one example of country-level ownership [75, 76]. Further considerations for financing social welfare systems include raising domestic tax revenues and reallocation of public funds for social protection programming.

### 5.2 Funding HIV Prevention Programmes

Despite the Global Fund’s calls for investments towards HIV prevention, funding for the HIV response in the ESAR has been on the decline and driven primarily by donor agendas. The ESAR, which is home to 50% of people living with HIV worldwide, obtains 55% of its HIV-related funding from international donors [54]. The region accounts for 82% of funding received directly from the United States of America under PEPFAR [54]. Other donor-supported HIV programmes for youth in the region include the DFID-Youth Agenda and SIDA-Investing in Future Generations [77].

To maximise impact, HIV treatment and combination prevention efforts must be complementary. With the increasing demand for HIV treatment, funding for HIV prevention is falling behind. Currently, only 20% of global resources for HIV programming are being spent on HIV prevention [77, 78]. Political leaders in the 2016 United Nations Political Declaration on Ending AIDS agreed that member states should be spending at least 25% of their total HIV budget on HIV prevention [79]. Investing more in HIV prevention is critical. UNAIDS modelling has revealed that investing around a quarter of all the resources required for the AIDS response in HIV prevention services would be sufficient to provide a range of HIV prevention programmes, including condom programmes, PrEP, VMMC, programmes to empower adolescent girls and young women, and mobilising key populations and providing them with essential service packages [80].

Data shows that donor contributions declined from US$8.62 billion in 2014 to US$7.53 billion in 2015, a 13% decline [81]. The US, which contributes the majority of funding towards combatting HIV (66%), decreased its financing from $5.6 billion in 2014 to $5 billion in 2015 [54, 82]. Notwithstanding the lack of precise data on funding directed towards HIV prevention among young people, the general reduction in HIV funding is likely to have a negative impact on access to HIV prevention services for young populations. The current decline in funding has occurred when there is still a wide gap in ensuring universal access to key HIV prevention services for young people in the ESAR, including the provision of age-appropriate education and essential sexual and reproductive health and treatment services [34]. This further contributes to the challenge of lowering HIV incidence and mortality rates [34, 82].

Efforts to meet the objective of ending the global HIV epidemic by 2030 requires sustained fiscal commitment. HIV funding needs to be addressed by framing a vision that focuses on mobilising local resources for evidence-based prevention interventions among young people. For HIV prevention interventions to be sustainable in the long-term (*e.g.* DREAMS), donor commitments need to be paralleled by country-level investments with local ownership.

Some countries (*e.g.*, South Africa and Namibia) significantly fund their HIV treatment programmes. [77, 83]. Other countries could also implement domestic financing initiatives, with one option being to institute an HIV tax/levy, similar to the one used in Zimbabwe [83]. Other innovative financing instruments that could be utilised include remittances and diaspora bonds, social and development impact bonds, sovereign wealth funds, and risk and credit guarantees [84]. Additionally, innovations could include public-private sector partnerships, surcharges on international calls and international flights [82]. In this environment, improving the targeting, efficiency, effectiveness and financial sustainability of HIV programmes is essential. Governments should ensure that each dollar invested achieves the highest return on investment and improved health outcomes. This can be done through optimised allocation of resources, public accountability of funds, and the implementation of cascades to monitor HIV programming.

### 5.3 Civil Society: Service Providers or Advocates for Legal Reform?

The single most effective way to reduce the financial burden of AIDS is to revitalise and scale-up HIV prevention initiatives [85]. The history of AIDS has shown the central importance of civil society organisations (CSOs) in shaping global, regional and national HIV responses, enabling accessible and cost-effective provision of services including treatment and PMTCT, as well as education in areas that are largely inaccessible to government. CSOs have also been instrumental in advocating for the rights of young people and LGBTI, and lobbying donors to fund the AIDS response [86]. CSOs are various associations, both formal and non-formal that represent the broad voluntary interests, purposes and ties that characterise a society [87]. Previous research has documented the influence of CSOs advocacy in developing health policies particularly in areas of influencing leadership, networking, credibility and resources [88].

Given the central importance of civil action in the HIV prevention response, the actions of CSOs are challenged on many fronts. In most cases, their work is considered apolitical, thereby, minimising chances of addressing from a policy change perspective [88-90]. Although governments tolerate advocacy, the participation of CSOs in policy and decision-making is mostly tokenistic and largely driven by libertarian service provision agendas, which makes long-term advocacy challenging [90]. Effective CSO policy engagement is also limited by government corruption, lack of openness to CSO engagement [89], and under-resourced CSOs with limited advocacy capacity. Further, a sense of fear and mistrust between CSOs and respective governments stifles the establishment of ‘state-society synergy’ [88, 89]. Consequently, a shift from advocacy towards service delivery is seen as a more comfortable option for some CSOs.

Given their history in promoting perspectives of vulnerable groups in national HIV and AIDS policies and programmes [90], an important role for CSOs is advocacy for legal reform. However, prohibitive laws (*e.g.*, age of consent for sexual activity, age of consent to medical treatment, criminalisation of consensual sexual activity between adolescents, criminalisation of HIV transmission) make advocacy for legal reform a complex and hostile space to work in.

Advancing the rights of marginalised and vulnerable populations in contexts where legislative and normative conditions prevent access to HIV and SRH services, care and support modalities is seen as a necessary component of an evolved HIV prevention response. An example of a regional NGO initiative is the Southern African Litigation Centre [91], which uses public interest litigation, training and advocacy to advance human rights in 11 southern African countries. Good practices of like-minded organisations include creating progressive jurisprudence, which advances human rights, instigates reform of national laws that do not comply with international human rights law, documenting human rights violations by the judiciary and enabling individuals to seek remedies for human rights violations. Clearly, there is a need for resources (fiscal and technical) to support communities to strengthen their engagement in policy-making and reform on removing legal barriers to accessing HIV and SRH services [92, 93].

## CONCLUSION

The global commitment to ending the AIDS epidemic by 2030 invariably places HIV prevention at the heart of the response. Reducing the new tide of infections in young populations in the ESAR is integral to the prevention response if this ambitious target is to be achieved. However, a number of challenges lie ahead. These challenges include gaps in epidemiological and behavioural profiles of young populations, complexities in HIV research with adolescents and young people, discriminatory (and contradictory) age-related legislature and policies on consent to sex, access to HIV testing and counselling and SRH services. Availability and sharing of information (*e.g.*, epidemic dynamics, programming successes and lessons learned) across sectors, countries and stakeholders, in an effective and timely manner, is a continuous challenge for effective programming. Furthermore, the rigid legislative and normative environments in the countries in the ESAR are major obstacles to accessing services for marginalised populations. While the science of HIV combination prevention offers hope, implementation and scale-up of HIV programmes remain challenges. The resources that are required to drive and sustain the HIV prevention response require effective cross-sector partnerships with a regional vision and country-led processes enabled through strong participation and accountability from all stakeholders.

## Figures and Tables

**Table 1:** Categorization of HIV prevention activities according to the HIV prevention cascade [46].

–	**Type of Intervention**	**Subcategory (if applicable)**
Demand side interventions	• Psycho-social and behavioural	• Young men and women, males who have sex with males, female sex workers, people who use drugs including alcohol
Supply side interventions	• Integration of HIV services • Condom distribution • Community level STI (sexually transmitted infections) interventions	• Young people, males who have sex with males, female sex workers, people who use drugs including alcohol
Adherence interventions	• Counselling • Socio-economic	• HIV testing and counselling, HIV positive prevention • Cash transfer interventions
Direct mechanisms of HIV prevention	• Voluntary medical male circumcision • Condom • PrEP (pre-exposure prophylaxis) • STI treatment • HIV treatment	• Males to females transmission, females to males transmission, males who have sex with males

## References

[1] https://www.unicef.org/esaro/theregion.html.

[2] https://data.unicef.org/wp-content/uploads/2017/11/HIVAIDS-Statistical-Update-2017.pdf.

[3] Bekker L.G., Johnson L., Wallace M., Hosek S. (2015). Building our youth for the future.. J. Int. AIDS Soc..

[4] https://www.reproductiverights.org/project/adolescents-access-to-reproductive-health-.

[5] Sawyer S.M., Afifi R.A., Bearinger L.H., Blakemore S.J., Dick B., Ezeh A.C., Patton G.C. (2012). Adolescence: A foundation for future health.. Lancet.

[6] UNAIDS Towards a global HIV prevention coalition and road map UNAIDS 2017; [cited 2017 December 12].. http://www.unaids.org/sites/default/files/media_asset/towards-global-HIV-prevention-coalition-and-road-map_en.pdf.

[7] UNICEF For every child, end AIDS: Seventh stocktaking report. UNICEF 2016; [cited 2017 December 12].. https://www.unicef.org/publications/index_93427.html.

[8] Buthelezi U.E., Davidson C.L., Kharsany A.B.M. (2016). Strengthening HIV surveillance: Measurements to track the epidemic in real time.. Afr. J. AIDS Res..

[9] Tanser F., Bärnighausen T., Grapsa E., Zaidi J., Newell M.L. (2013). High coverage of ART associated with decline in risk of HIV acquisition in rural KwaZulu-Natal, South Africa.. Science.

[10] UNICEF UNICEF annual report 2013: Botswana. UNICEF 2013; [cited 2017 December 12].. https://www.unicef.org/about/annualreport/files/Botswana_COAR_2013.pdf.

[11] Ministry of Health [Lesotho], ICF International. Lesotho demographic and health survey 2014. Ministry of Health [Lesotho] 2016; [cited 2017 December 12].. https://dhsprogram.com/pubs/pdf/FR309/FR309.pdf.

[12] Kenya National Bureau of Statistics, Ministry of Health [Kenya], National AIDS Control Council, Kenya Medical Research Institute, National Council for Population and Development, ICF International. Kenya demographic and health survey 2014. Kenya National Bureau of Statistics 2015; [cited 2017 December 12].. https://dhsprogram.com/pubs/pdf/fr308/fr308.pdf.

[13] Central Statistical Office [Zambia], Ministry of Health [Zambia], ICF International Zambia demographic and health survey 2013-14 Central Statistical Office [Zambia], Ministry of Health [Zambia], and ICF International 2014; [cited 2017 December 12].. https://www.dhsprogram.com/pubs/pdf/FR304/FR304.pdf.

[14] National Statistical Office [Malawi], ICF International. Malawi demographic and health survey 2015-16. National Statistical Office [Malawi] and ICF International 2017; [cited 2017 December 12].. http://www.dhsprogram.com/pubs/pdf/MIS18/MIS18.pdf.

[15] http://www.unaids.org/sites/default/files/media_asset/UNAIDS_HIV_prevention_among_adolescent_girls_and_young_women.pdf.

[16] UNICEF Gender and HIV/AIDS: prevention among young people. UNICEF 2016; [cited 2017 December 12].. https://www.unicef.org/esaro/7310_Gender_HIV_prevention_among_youth.html.

[17] Evan M., Risher K., Zungu N., Shisana O., Moyo S., Celentano D.D., Maughan-Brown B., Rehle T.M. (2016). Age-disparate sex and HIV risk for young women from 2002 to 2012 in South Africa.. J. Int. AIDS Soc..

[18] de Oliveira T., Kharsany A.B., Gräf T., Cawood C., Khanyile D., Grobler A., Puren A., Madurai S., Baxter C., Karim Q.A., Karim S.S. (2017). Transmission networks and risk of HIV infection in KwaZulu-Natal, South Africa: A community-wide phylogenetic study.. Lancet HIV.

[19] Harling G., Newell M.L., Tanser G., Harling F., Kawachi I., Subramanian S.V., Bärnighausen T. (2014). Do age-disparate relationships drive HIV incidence in young women? evidence from a population cohort in rural KwaZulu-Natal, South Africa.. J Acquir Immune Def Syndr.

[20] Conner B. (2015). First, do no harm: Legal guidelines for health programmes affecting adolescents aged 10-17 who sell sex or inject drugs.. J. Int. AIDS Soc..

[21] World Health Organization Global accelerated action for the health of adolescents (AA-HA!); Guidance to support country implementation –summary. World Health Organisation 2017; [cited 2018 May 23].. http://africahealthforum.afro.who.int/IMG/pdf/the_global_accelerated_action_for_the_health_of_adolescent_aa-ha_implementation_guidance.pdf.

[22] Delany-Moretlwe S., Cowan F.M., Busza J., Bolton-Moore C., Kelley K., Fairlie L. (2015). Providing comprehensive health services for young key populations: needs, barriers and gaps.. J. Int. AIDS Soc..

[23] Weldegebreal R., Melaku Y.A., Alemayehu M., Gebrehiwot T.G. (2015). Unintended pregnancy among female sex workers in Mekelle city, northern Ethiopia: A cross-sectional study.. BMC Public Health.

[24] Boonzaier F., Zway M. (2015). Young lesbian and bisexual women resisting discrimination and negotiating safety: A photovoice study: Original
contributions.. Afr Saf Promot.

[25] Wolff N., Blitz C.L., Shi J., Bachman R., Siegel J.A. (2006). Sexual violence inside prisons: Rates of victimization.. J. Urban Health.

[26] UNICEF Thematic discussion on the work of UNICEF in humanitarian situations. UNICEF 2012; [cited 2017 December 12].. https://www.unicef.org/about/execboard/files/2012-CRP28-Thematic_discussion_on_Humanitarian_Action_FINAL_8Aug2012.pdf.

[27] Pettifor A., Nguyen N.L., Celum C., Cowan F.M., Go V., Hightow-Weidman L. (2015). Tailored combination prevention packages and PrEP for young key populations.. J Int AIDS Soc.

[28] Higgins J.A., Hoffman S., Dworkin S.L. (2010). Rethinking gender, heterosexual men, and women’s vulnerability to HIV/AIDS.. Am. J. Public Health.

[29] http://www.unaids.org/sites/default/files/media_asset/JC2848_en.pdf.

[30] Lindegger G., Quayle M.

[31] Govender K. (2011). The cool, the bad, the ugly, and the powerful: Identity struggles in schoolboy peer culture.. Cult. Health Sex..

[32] Jewkes R., Morrell R., Hearn J., Lundqvist E., Blackbeard D., Lindegger G., Gottzén L. (2015). Hegemonic masculinity: Combining theory and practice in gender interventions.. Cult Health Sex.

[33] Mills E. J., Beyrer C., Birungi J., Dybul M. R. (2012). Engaging men in prevention and care for HIV/AIDS in Africa.. PLoS Med.

[34] UNAIDS Prevention gap report. UNAIDS 2016; [cited 2017 December 12].. http://www.unaids.org/sites/default/files/media_asset/2016-prevention-gap-report_en.pdf.

[35] Barker G., Greene M., Siegel E. G. https://www.icrw.org/wp-content/uploads/2016/10/What-Men-Have-to-Do-With-It.pdf.

[36] http://www.icad-cisd.com/pdf/Gender_Issues_EN_FINAL.pdf.

[37] Gibbs A., Jewkes R., Sikweyiya Y., Willan S. (2015). Reconstructing masculinity? a qualitative evaluation of the Stepping Stones and Creating Futures interventions in urban informal settlements in South Africa.. Cult Health Sex.

[38] Gibbs A., Vaughan C., Aggleton P. (2015). Beyond ‘working with men and boys’: (re) Defining, challenging and transforming masculinities in sexuality and health programmes and policy.. Cult. Health Sex..

[39] Horwood C., Butler L.M., Haskins L., Phakathi S., Rollins N. (2013). HIV-infected adolescent mothers and their infants: Low coverage of HIV services and high risk of HIV transmission in KwaZulu-Natal, Durban, South Africa.. PLoS One.

[40] Kaufman M.R., Smelyanskaya M., Van Lith L.M., Mallalieu E.C., Waxman A., Hatzhold K., Marcell A.V., Kasedde S., Lija G., Hasen N., Ncube G., Samuelson J.L., Bonnecwe C., Seifert-Ahanda K., Njeuhmeli E., Tobian A.A. (2016). Adolescent sexual and reproductive health services and implications for the provision of voluntary medical male circumcision: results of a systematic literature review.. PLoS One.

[41] Govender K., George G., Beckett S., Montague C., Frohlich J. (2017). Risk compensation following medical male circumcision: results from a 1-
year prospective cohort study of young school-going men in KwaZulu-Natal, Durban, South Africa.. Int. J. Behav. Med..

[42] Njeuhmeli E., Hatzold K., Gold E., Mahler H., Kripke K., Seifert-Ahanda K., Castor D., Mavhu W., Mugurungi O., Ncube G., Koshuma S., Sgaier S., Conly S.R., Kasedde S. (2014). Lessons learned from scale-up of voluntary medical male circumcision focusing on adolescents: Benefits, challenges, and potential opportunities for linkages with adolescent HIV, sexual, and reproductive health services.. J Acquir Immune Defic Syndr.

[43] Baxter C., Abdool Karim S. (2016). Combination HIV prevention options for young women in Africa.. Afr. J. AIDS Res..

[44] Harrison A., Hoffman S., Mantell J.E., Smit J.A., Leu C.S., Exner T.M., Stein Z.A. (2016). Gender-focused HIV and pregnancy prevention for school-going adolescents: The Mpondombili pilot intervention in KwaZulu-Natal, South Africa.. J HIV AIDS Soc Serv.

[45] Kaiser R., Bunnell R., Hightower A., Kim A.A., Cherutich P., Mwangi M., Oluoch T., Dadabhai S., Mureithi P., Mugo N., Mermin J., KAIS Study Group (2011). Factors associated with HIV infection in married or cohabitating couples in Kenya: Results from a nationally representative study.. PLoS One.

[46] Godfrey-Faussett P. (2016). The HIV prevention cascade: More smoke than thunder?. Lancet HIV.

[47] Hargreaves J.R., Delany-Moretlwe S., Hallett T.B., Johnson S., Kapiga S., Bhattacharjee P., Dallabetta G., Garnett G.P. (2016). The HIV prevention cascade: Integrating theories of epidemiological, behavioural, and social science into programme design and monitoring.. Lancet HIV.

[48] Krishnaratne S., Hensen B., Cordes J., Enstone J., Hargreaves J.R. (2016). Interventions to strengthen the HIV prevention cascade: A systematic review of reviews.. Lancet HIV.

[49] Isbell M.T., Kilonzo N., Mugurungi O., Bekker L.G. (2016). We neglect primary HIV prevention at our peril.. Lancet HIV.

[50] Fauci A.S., Folkers G.K., Dieffenbach C.W. (2013). HIV-AIDS: Much accomplished, much to do.. Nat. Immunol..

[51] Krakower D.S., Mayer K.H. (2015). Pre-exposure prophylaxis to prevent HIV infection: Current status, future opportunities and challenges.. Drugs.

[52] Gengiah T.N., Moosa A., Naidoo A., Mansoor L.E. (2014). Adherence challenges with drugs for pre-exposure prophylaxis to prevent HIV infection.. Int. J. Clin. Pharm..

[53] https://www.avac.org/resource/2012-avac-report-achieving-end%E2%80%94one-year-and-counting.

[54] https://www.pepfar.gov/partnerships/ppp/dreams/.

[55] Peterson I., Govender K., Peterson I., Bhana A., Flisher A., Swartz L., Richter L. (2010). Theoretical considerations: From understanding to intervening.. Promoting Mental Health in Scarce Resource Contexts..

[56] https://www.unicef.org/eapro/Lost_in_Transitions.pdf.

[57] Hosek S.G., Zimet G.D. (2010). Behavioral considerations for engaging youth in HIV clinical research.. J Acquir Immune Defic Syndr.

[58] Flicker S., Guta A. (2008). Ethical approaches to adolescent participation in sexual health research.. J. Adolesc. Health.

[59] International Planned Parenthood Federation Sexual and reproductive rights of young people: Autonomous decision making and confidential services. International Planned Parenthood Federation 2012; [cited 2017 December 12].. https://www.ippfwhr.org/sites/default/files/srrightsyoungen.pdf.

[60] United Nations Population Fund Harmonizing the legal environment for adolescent sexual and reproductive health and rights: A review of 23 countries in East and Southern Africa. United Nations Population Fund 2017; [cited 2017 December 12].. http://esaro.unfpa.org/en/publications/harmonizing-legal-environment-adolescent-sexual-and-reproductive-health-and-rights-0.

[61] Sarumi R.O., Strode A.E. (2015). New law on HIV testing in Botswana: The implications for healthcare professionals.. South. Afr. J. HIV Med..

[62] Fischer S., Reynolds H., Yacobson I., Barnett B., Schueller J. HIV counselling and testing for youth: A manual for providers. Family Health International 2007; [cited 2017 December 12].. https://www.fhi360.org/sites/default/files/media/documents/HIV%20Counseling%20and%20Testing%20for%20Youth.pdf.

[63] https://eduinfoafrica.files.wordpress.com/2016/11/basiceducationrightshandbook-complete.pdf.

[64] http://data.unaids.org/pub/BaseDocument/2010/jc1879_social_protection_business_case_en.pdf.

[65] UNICEF, Eastern and Southern Africa Regional Office The impact of social cash transfers on children affected by HIV and AIDS: Evidence from Zambia, Malawi and South Africa, UNICEF, Eastern and Southern Africa Regional Office 2007; [cited 2017 December 12].. http://docplayer.net/41353990-The-impact-of-social-cash-transfers-on-children-affected-by-hiv-and-aids-evidence-from-zambia-malawiand-south-africa.html.

[66] Handa S., Halpern C.T., Pettifor A., Thirumurthy H. (2014). The government of Kenya’s cash transfer program reduces the risk of sexual debut among young people age 15-25.. PLoS One.

[67] Hallfors D., Cho H., Rusakaniko S., Iritani B., Mapfumo J., Halpern C. (2011). Supporting adolescent orphan girls to stay in school as HIV risk prevention: evidence from a randomized controlled trial in Zimbabwe.. Am. J. Public Health.

[68] Taaffe J., Cheikh N., Wilson D. (2016). The use of cash transfers for HIV prevention--are we there yet?. Afr. J. AIDS Res..

[69] Cluver L.D., Orkin F.M., Meinck F., Boyes M.E., Yakubovich A.R., Sherr L. (2016). Can social protection improve sustainable development goals for adolescent health?. PLoS One.

[70] Cluver L.D., Orkin F.M., Yakubovich A.R., Sherr L. (2016). Combination social protection for reducing HIV-risk behaviour amongst adolescents in South Africa.. J. Acquir. Immune Defic. Syndr..

[71] Toska E., Cluver L.D., Boyes M.E., Isaacsohn M., Hodes R., Sherr L. (2017). School, supervision and adolescent-sensitive clinic care: Combination social protection and reduced unprotected sex among HIV positive adolescents in South Africa.. AIDS Behav..

[72] Toska E., Gittings L., Hodes R., Cluver L.D., Govender K., Chademana K.E., Gutiérrez V.E. (2016). Resourcing resilience: Social protection for HIV prevention amongst children and adolescents in Eastern and Southern Africa.. Afr. J. AIDS Res..

[73] United Nations Development Programme Social protection for sustainable development: dialogues between Africa and Brazil. United Nations Development Programme 2016; [cited 2017 December 12].. http://www.undp.org/content/undp/en/home/librarypage/poverty-reduction/social-protection-for-sustainable-development--dialogues-between.html.

[74] Atun R., Silva S., Ncube M., Vassall A. (2012). Innovative financing for HIV response in sub-Saharan Africa.. J Glob Health.

[75] Nyamukapa H. (2016). Cash transfers and early childhood care and education in Zimbabwe: A critical inquiry to discourse, theory and practice.. S Afr J Child Educ.

[76] UNICEF Annual results report. UNICEF 2015; [cited 2017 December 12].. https://www.unicef.org/publicpartnerships/files/2015ARR_HIVAIDS.pdf.

[77] Resch S., Ryckman T., Hecht R. (2015). Funding AIDS programmes in the era of shared responsibility: an analysis of domestic spending in 12 low-income and middle-income countries.. Lancet Glob. Health.

[78] UNAIDS Press release. UNICEF 2016; [cited 2017 December 12].. http://www.unaids.org/en/resources/presscentre/pressreleaseandstatementarchive/2016/july/20160662_prevention-gap.

[79] UNAIDS Fast-track update on investments needed in the AIDS response. UNAIDS 2016; [cited 2017 December 12].. http://www.unaids.org/sites/default/files/media_asset/UNAIDS_Reference_FastTrack_Update_on_investments_en.pdf.

[80] UNAIDS Invest in HIV prevention. UNAIDS 2015; [cited 2017 December 12].. http://www.unaids.org/sites/default/files/media_asset/JC2791_invest-in-HIV-prevention_en.pdf.

[81] UNAIDS Financing the response to HIV in low and middle-income countries. UNAIDS 2016; [cited 2017 December 12].. http://www.unaids.org/sites/default/files/media_asset/financing-the-response-to-HIV-in-low-and-middle-income-countries_en.pdf.

[82] https://www.cabsa.org.za/Show-Me-Money-Global-HIVAIDS-Funding-Decline-2272016.

[83] UNAIDS Smart investments. UNAIDS 2013; [cited 2017 December 12].. http://www.unaids.org/en/media/unaids/contentassets/documents/unaidspublication/2013/20131130_smart-investments_en.pdf.

[84] Atun R., Silva S., Ncube M. (2016). Innovative financing for HIV response in sub-Saharan Africa.. J Glob Health.

[85] Poku N.K., Bonnel R., Ncube M. (2016). Funding of community-based interventions for HIV prevention.. Afri J AIDS Res.

[86] https://www.unicef-irc.org/files/documents/d-3827-HIV-and-Social-Protection.pdf.

[87] OECD Glossary of statistical terms: civil society organizations. OECD 2007; [cited 2017 December 12].. https://stats.oecd.org/glossary/detail.asp?ID=7231.

[88] Court M., Mendizabal E., Osborne D., Young J. Policy engagement: How civil society can be more effective. Overseas Development Institute 2006; [cited 2017 December 12].

[89] Williamson R.T., Rodd J. (2016). Civil society advocacy in Nigeria: promoting democratic norms or donor demands?. BMC Int. Health Hum. Rights.

[90] Spicer N., Harmer A., Aleshkina J., Bogdan D., Chkhatarashvili K., Murzalieva G., Rukhadze N., Samiev A., Walt G. (2011). Circus monkeys or change agents? civil society advocacy for HIV/AIDS in adverse policy environments.. Soc Sci Med.

[91] https://southernafricalitigationcentre.org/annual-reports-and-newsletters/.

[92] Loewenson R. Civil society-state interactions in national health systems. World Health Organization and Training and Research Support Centre [Zimbabwe] 2003; [cited 2017 December 12].. http://tarsc.org/WHOCSI/pdf/WHOTARSC2.pdf.

[93] Hushie M., Omenyo C.N., van den Berg J.J., Lally M.A. (2016). State-civil society partnerships for HIV/AIDS treatment and prevention in Ghana: Exploring factors associated with successes and challenges.. BMC Health Serv. Res..

